# Expression and gene regulation network of *INHBA* in Head and neck squamous cell carcinoma based on data mining

**DOI:** 10.1038/s41598-019-50865-y

**Published:** 2019-10-04

**Authors:** Zeng-hong Wu, Yun Tang, Xun Niu, Qing Cheng

**Affiliations:** 1 Department of Otorhinolaryngology, Union Hospital, Tongji Medical College, Huazhong University of Science and Technology, Wuhan, Hubei China; 2 Department of Infectious Diseases, Union Hospital, Tongji Medical College, Huazhong University of Science and Technology, Wuhan, 430022 China; 3 Department of Critical Care Medicine, Union Hospital, Tongji Medical College, Huazhong University of Science and Technology, Wuhan, 430022 China

**Keywords:** Gene expression, Cancer genomics

## Abstract

Inhibin subunit beta A(INHBA) encodes an individual from the TGF-β superfamily of proteins and the ligand could be further homo-dimerized to shape activin A or hetero-dimerized to frame inhibin with inhibin beta B. We studied INHBA expression, mutations, regulation, function networks and immune infiltrates in data from patients with Head and neck squamous cell carcinoma (HNSCC) based on different open databases by utilizing multi-dimensional investigation techniques. This study gives staggered evidence for the significance of INHBA in head and neck squamous cell carcinoma and its potential role as a novel biomarker. Our outcomes propose that INHBA overexpression in HNSCC has profound impacts in the center hub of post-transcriptional regulation, which is firmly identified with protein translation. Meanwhile, we also examine the function of the identified miRNAs that were related to INHBA and molecular function of these miRNAs were mainly enhanced in transcription factor activity, transcription regulator activity. In addition, B cells of immune infiltrates affecting the prognosis and might have a prognostic significance related to INHBA in HNSCC. Our outcomes show that data mining efficiently uncovers information about INHBA expression in HNSCC and more importance establishing a foundation for further investigation of the role of INHBA in carcinogenesis.

## Introduction

Head and neck squamous cell carcinoma (HNSCC), a common malignant tumor of the head and neck distinct, which arises from lip, oral cavity, paranasal sinuses, oropharynx, larynx, nasopharynx and other pharynx carcinomas^1^. As the sixth most common type of malignant tumor with an incidence of over 650 000 new cases and a 90 000 deaths per year worldwide^2^. Currently, cigarette smoking, alcohol consumption as well as human papilloma virus (HPV) infection are deemed to be risk factors for the occurrence and prognosis of HNSCC^3^. Unfortunately, due to lack of symptoms in the early stage when detected of HNSCC is usually made at advanced stages and the 5-year survival rate is still under 50% now, while due to local recurrence and metastasis, which reduces survival rate to 35%^4^. The occurrences and progression of HNSCC is a complicated process involving multiple molecules. Guan *et al*. found long non-coding RNA H19 and its mature item miR-675 were significantly overexpressed in two HNSCC cell lines and a cohort of 65 primary tumor samples^5^. Wu *et al*. reported that SUZ12 protein was especially up-regulated in primary HNSCC samples and is overexpression significantly related to cervical node metastasis and decreased overall and disease-free survival^6^. Reed *et al*. suggested that inactivation of the p16 tumor suppressor gene is a frequent event in HNSCC^7^. Trivedi *et al*. suggested a link between the expression of several tumor markers (including parathyroid hormone-related peptide and pemphigus vulgaris antigen) and the metastasis of HNSCC to the lymph nodes^8^. Due to the histological types and multiple anatomical sites and of HNSCC the tumor markers vary widely; thus, it may be possible to identify of a more valuable drug targets for HNSCC by screening gene function networks for alters related to tumor formation and progression.

Inhibin subunit beta A (INHBA), otherwise called EDF or FRP, encodes an individual from the TGF-beta (transforming growth factor-beta) superfamily of proteins. Studies have reported that INHBA is up-regulated in gastric cancer and linked to poor survival, INHBA gene silencing can gives a potential target in the treatment of gastric cancer^9–11^. Seder *et al*. indicated that INHBA may be regulated by DNA methylation in lung adenocarcinoma^12^. In addition, abnormal overexpression of INHBA was detected in various malignant tumors, including esophageal cancer, prostate cancer, and ovarian cancer^13–15^. It was also discovered that INHBA is involved in tumor–node–metastasis (TNM) stage and venous invasion^16^. Wang *et al*. discovered that INHBA was linked to cancer diameter and tumor invasion depth, and further suggested that patients with a higher expression of INHBA had a shorter disease‐free survival rate^17^. Above these results propose that INHBA may be a novel proto-oncogene. However, determining the interaction between INHBA and HNSCC remains an unsolved problem. Thus, in the present study our objection is to studied INHBA expression, mutations, regulation, function networks and immune infiltrates in data from patients with HNSCC based on different open databases by utilizing multi-dimensional analysis strategies. Our results will be of great significance in clarifying the pathogenesis of HNSCC and in filtering biomarkers for diagnosis.

## Results

### INHBA expression in HNSCC

**Figure 1:**
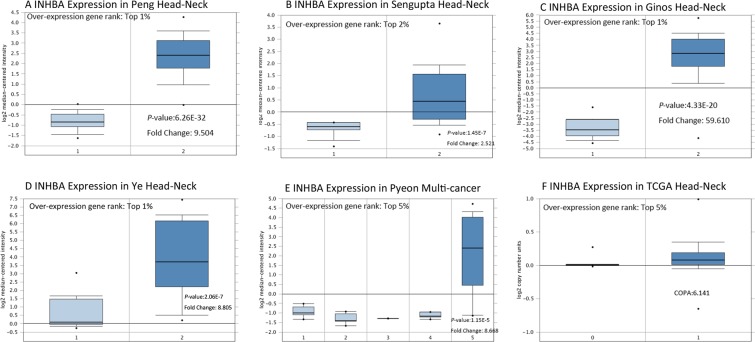
*INHBA* transcription in Head and Neck Cancer (Oncomine). Levels of *INHBA* mRNA and DNA copy number were significantly higher in Head and Neck Cancer than in normal tissue. Shown are fold change, associated *p* values, and overexpression gene rank, based on Oncomine 4.5 analysis. (**A**–**E**) Box plot showing *INHBA* mRNA levels in, respectively, the Peng Head-Neck, Sengupta Head-Neck, Ginos Head-Neck, Ye Head-Neck and Pyeon Multi-cancer Head-Neck datasets. F Box plot showing *INHBA* copy number in The Cancer Genome Atlas (TCGA) Head and Neck.

**Figure 2:**
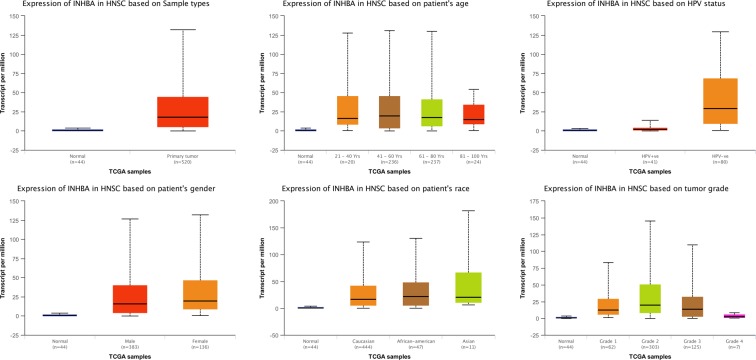
Boxplot showing relative expression of *INHBA* in subgroups of patients with Head and Neck Cancer, stratified based on gender, age, HPV status, gender, race and tumor grade (UALCAN). *P* < 0.05.

Information in the Oncomine database uncovered that mRNA expression and DNA copy number variation of INHBA were fundamentally higher in HNSCC tissues when compare with normal tissues (*P* < 0.01). Box plot showing *INHBA* mRNA levels in, respectively, the Peng Head-Neck, Sengupta Head-Neck, Ginos Head-Neck, Ye Head-Neck and Pyeon Multi-cancer Head-Neck datasets, meanwhile, the fold change differences all were over 2 means that INHBA expression were significantly higher in HNSCC tissues **(**Fig. 1). Further subgroup analysis of multiple clinic pathological features of 520 HNSCC samples in the TCGA reliably indicated high transcription of INHBA. In age subgroup (normal-vs-age (21–40 yrs), normal-vs-age (41–60 yrs), normal-vs-age (61–80 yrs) and normal-vs-age (81–100 yrs)) analysis the transcription level of INHBA was essentially higher in HNSCC patients than healthy individuals. In HPV status subgroups (normal-vs-HPV − ve and normal-vs-HPV + ve) analysis; gender subgroup (normal-vs-male and normal-vs-female); race subgroup (normal-vs-Caucasian,normal-vs-AfricanAmerican and normal-vs-Asian); tumor grade subgroup (normal-vs-Grade 1, normal-vs-Grade 2, normal-vs-Grade 3 andnormal-vs-Grade 4) analysis the INHBA was also significantly higher in HNSCC patients (Fig. 2).

### Frequency and type of INHBA alterations in HNSCC

**Figure 3:**
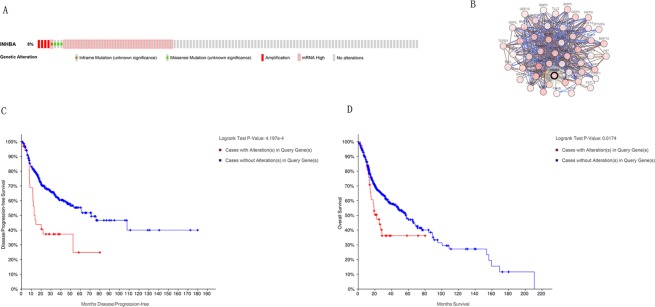
Visual summary of *INHBA* alterations and biological interaction network in Head and Neck Cancer (cBioPortal). (**A**) OncoPrint of *INHBA* alterations in HNSCC. (**B**) Network view of the *INHBA* neighborhood in HNSC. Darker red indicates increased frequency of alteration in HNSCC. Nodes with bold black outline represent hub genes. Nodes with thin black outline represent the co-expression genes. (**C**) Disease/Progression-free survival. *P* < 0.05. (**D**) Overall survival. *P* < 0.05.

We then based on cBioPortal to explore the sorts and frequency of INHBA modifications in HNSCC from HNSCC patients sequencing data in the TCGA database. INHBA was modified in 42 of 496 (8%) HNSCC patients (Fig. 3A). These modifications were mRNA upregulation in 34 cases, amplification in 4 cases, missense mutation in 3 cases and inframe mutation in 1 case. Thus, mRNA upregulation is the most common type of INHBA in HNSCC patients. Besides, Kaplan-Meier survival analysis demonstrated statistically significant that INHBA overexpression was related to overall survival (*P* < 0.05) and disease/progression-free survival (*P* < 0.05) in HNSCC (Fig. 3C,D).

### Biological interaction network of RBM8A

**Figure 4:**
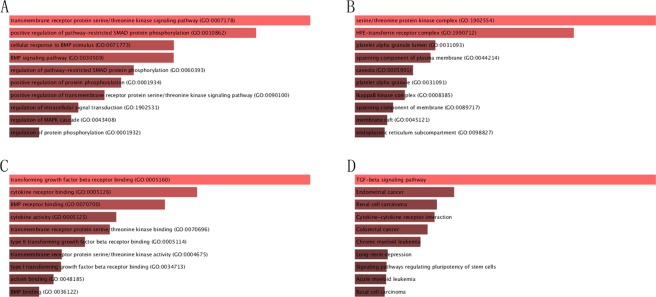
Enrichment analysis of the genes altered in the *INHBA* neighborhood in Head and Neck Cancer (Enrich). The bubble diagrams display the enrichment results of the top 50 genes altered in the *INHBA* neighborhood in HNSCC. (**A**) Biological processes. (**B**) Cellular components. (**C**) Molecular functions. (**D**) KEGG pathway analysis.

In order to decide the biological interaction network of INHBA in HNSCC, we applied to tab Network in cBioPortal to demonstrate INHBA neighboring genes that were changed at top 10 frequencies (Fig. 3B and Table 1). The results indicated that the most amplification gene were GDNF and GDF6; the most up-regulation and down-regulation gene was FKBP1A and MAP3K7, respectively; the most mutation gene were HRAS and the most total alteration was GDNF (Table 1). The 50 most as often altered neighbor genes of INHBA were showed utilizing Network and the most frequent alterations were GDNF (13.1%), GDF6 (10.9%) and MAP3K7 (10.1%). To examine the function of the identified 50 neighbor genes, biological analyses were performed utilizing GO enrichment and KEGG pathway analysis via Enrichr online database. *P* < 0.05 as the criterion deemed statistically significant. GO analysis results demonstrated that biological processes (BP) were significantly enriched in transmembrane receptor protein serine/threonine kinase signaling pathway, positive guideline of pathway-restricted SMAD protein phosphorylation, cellular response to BMP stimulus *et al*. Molecular function (MF) were mainly enhanced in activing binding, ATP binding, BMP binding *et al*. Cell component (CC) were primarily enriched in the serine/threonine protein kinase complex, HFE-transferrin receptor complex, membrane raft *et al*. KEGG analysis revealed that the most part enriched in TGF-beta signaling pathway, Endometrial cancer, Renal cell carcinoma *et al*. **(**Fig. 4). Therefore, the biological interaction network of INHBA alterations is engaged with the activing binding, protein complex form, regulation of protein and several cancer processes.

**Table 1:** The type and frequency of *INHBA* neighbor gene alterations in HNSCC (cBioPortal).

Gene Symbol	Amplification	Homozygous Deletion	Up-regulation	Down-regulation	Mutation	Total Alteration
INHBA	0.8	0	7.1	0	0.8	8.5
VCVR1	0.6	0.2	6.5	0	0	8.1
VCVR1B	0	0	4.4	1.4	1.0	6.9
BMP1	0	2.8	6.5	0	0.6	9.7
GDNF	4.6	0	8.3	0	0.8	13.1
MAP3K7	0.4	0.2	4.4	3.8	1.6	10.1
HRAS	0.6	0.4	3.6	0	6	9.3
GDF6	4.6	0	5.2	0	1.2	10.9
BMPS	2.8	0	4	0	1.2	7.5
FKBP1A	0.4	0.2	9.1	0	0	9.5

### GO and KEGG pathway examinations of co-expression genes connected with INHBA in HNSCC

**Figure 5:**
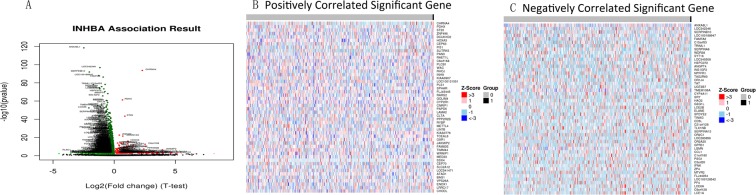
Genes differentially expressed in correlation with *INHBA* in Head and Neck Cancer (LinkedOmics). (**A**) A T-test was used to analyze correlations between *INHBA* and genes differentially expressed in HNSCC. (**B**,**C**) Heat maps showing genes positively and negatively correlated with *INHBA* in HNSCC (TOP 50). Red indicates positively correlated genes and blue indicates negatively correlated genes.

**Figure 6:**
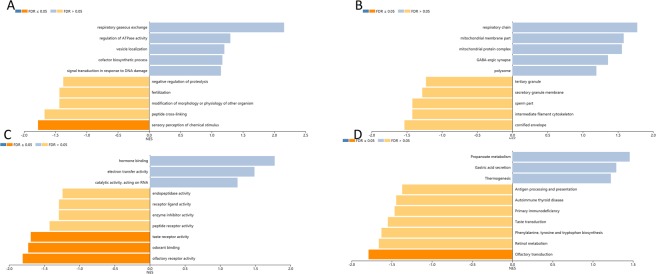
Significantly enriched GO annotations and KEGG pathways of *INHBA* in Head and Neck Cancer. The significantly enriched GO annotations and KEGG pathways of *INHBA* co-expression genes in HNSCC were analyzed using GSEA. (**A**) Biological processes. (**B**) Cellular components. (**C**) Molecular functions. (**D**) KEGG pathway analysis.

LinkedOmics was utilized to analyze mRNA sequencing information from HNSCC patients in the TCGA. A T-test was utilized to analyze connections among *INHBA* and genes differentially expressed in HNSC (Fig. 5A). The 50 critical gene sets positively and negatively connected with INHBA as appeared in the heat map (Fig. 5B,C). Significant GO and KEGG term examination by gene set enrichment analysis (GSEA) demonstrated that genes differentially expressed in connection with INHBA were found essentially in the sensory perception of chemical stimulus, olfactory receptor activity and olfactory transduction (Fig. 6). We also list the 5 most significant target networks miRNA, kinase and transcription factor target networks of decidedly related INHBA created by GSEA and the results indicated that the most leading-edge number in kinase target, miRNA target and transcription factor target was kinase_GRK3, GTGCCAA, MIR-96 and V$CP2_01, respectively (Table 2).

**Table 2:** The Kinase, miRNA and transcription factor-target networks of *INHBA* in HNSCC (LinkedOmics).

Enriched Category	Geneset	Leading Edge Num
Kinase Target	Kinase_GRK3	279
	Kinase_CSNK1E	5
	Kinase_PDK1	1
	Kinase_MAP4K1	3
	Kinase_NTRK2	1
miRNA Target	TACTTGA,MIR-26A,MIR-26B	46
	AGGGCCA,MIR-328	25
	GTACTGT,MIR-101	70
	GTCTTCC,MIR-7	30
	GTGCCAA,MIR-96	86
Transcription Factor Target	V$WHN_B	59
	YNGTTNNNATT	64
	V$ALPHACP1_01	30
	V$CP2_01	70
	V$ARNT_02	64

Abbreviations: Leading Edge Num, the number of leading edge genes; V$, the annotation found in Molecular Signatures Database (MSigDB) for transcription factors (TF).

### miRNAs related to INHBA

**Figure 7:**
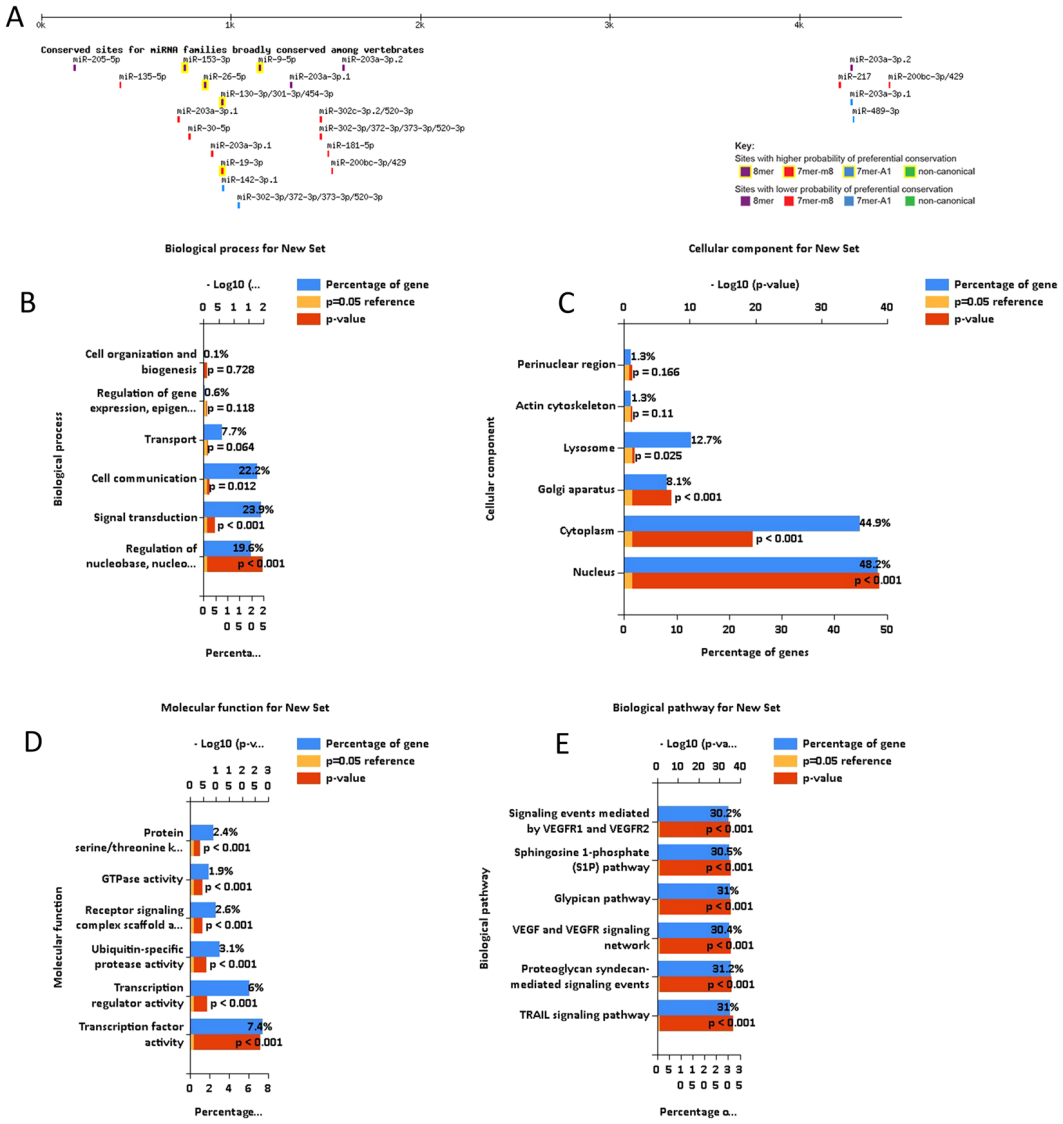
Enrichment analysis of the miRNA altered in the *INHBA* in Head and Neck Cancer (Funrich and Targetscan). (**A**) Conserved sites for miRNA families broadly conserved among vertebrates (**B**) Biological processes. (**C**) Cellular components. (**D**) Molecular functions. (**E**) KEGG pathway analysis.

According to cumulative weighted context++ score, the top 5 among 961 miRNAs family was miR-133a-3p.2/133b, miR-130-3p/301-3p/454-3p, miR-153-3p, miR-140-3p and miR-203a-3p.1 that related to gene INHBA. Conserved sites for miRNA family broadly conserved among vertebrates showed in Fig. 7A. To examine the function of the identified 961 miRNAs, biological enrichment was performed via Funrich database. Biological processes were significantly enriched in regulation of nucleic acid metabolism, cell communication and signal transduction; Cell component were primarily enriched in the nucleus, cytoplasm, golgi apparatus; Molecular function were mainly enhanced in ubiquitin-specific protease activity, transcription factor activity, transcription regulator activity, Biological pathway enriched in TRAIL signaling pathway, VEGY and VEGFR signaling pathway, glypican pathway.

### Immune infiltrates in correlation with INHBA in HNSCC

**Figure 8:**
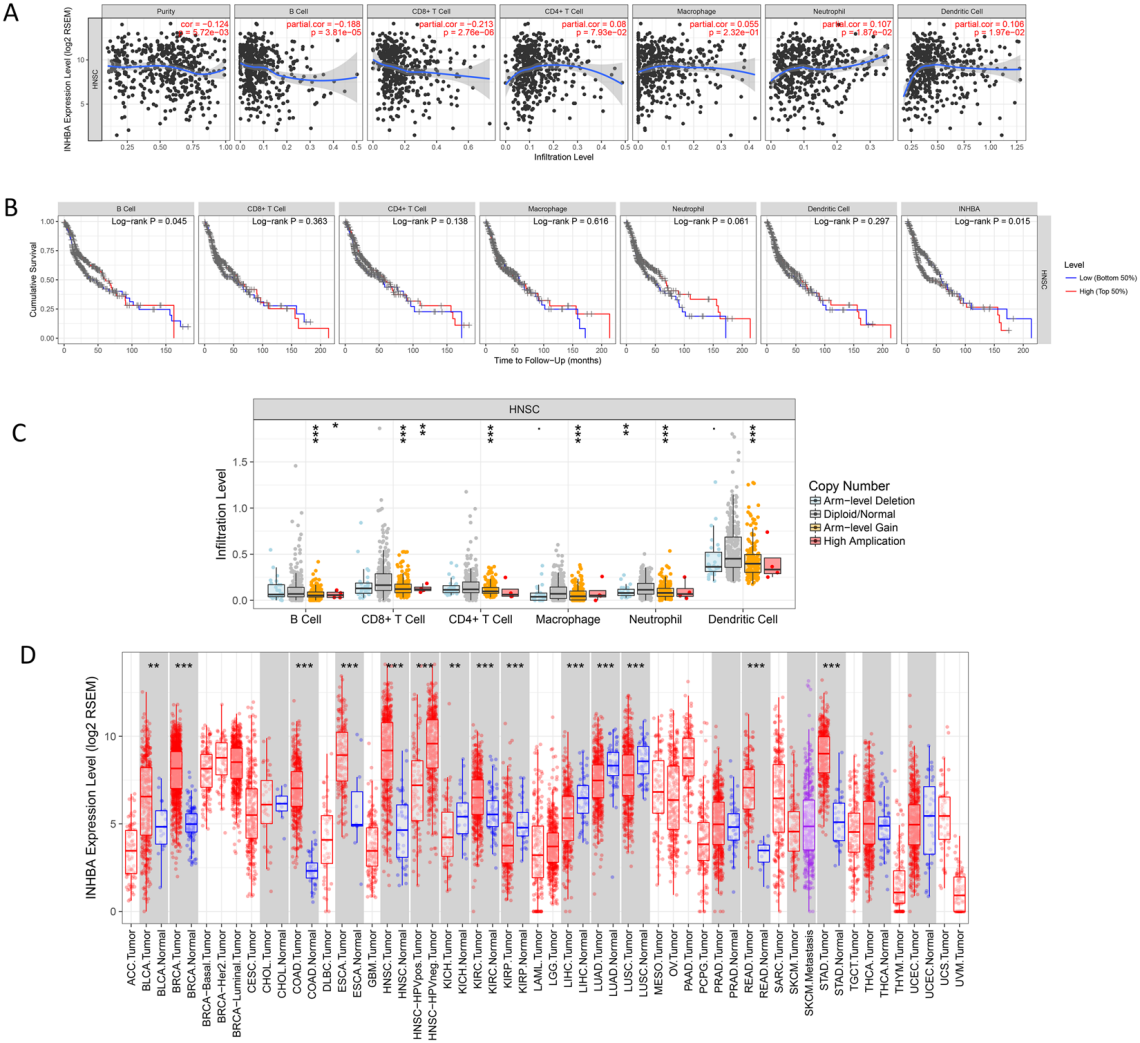
Immune infiltrates in correlation with *INHBA* in B cells, CD4+ T cells, CD8+ T cells, Neutrphils, Macrophages and Dendritic cells of Head and Neck Cancer (TIMER). (**A**) Correlation between *INHBA* expression and abundance of immune infiltrates. (**B**) Clinical outcome and abundance of immune infiltrates of *INHBA* expression. (**C**) Correlation between somatic copy number alterations (SCAN) and abundance of immune infiltrates of *INHBA*. (**D**) *INHBA* expression between tumor and normal tissue. *P*-value Significant Codes: 0 ≤ *** < 0.001 ≤ ** < 0.01 ≤ * < 0.05 ≤ . < 0.1.

Correlation between INHBA in HNSCC expression and abundance of immune infiltrates (B cells, CD4 + T cells, CD8 + T cells, Neutrphils, Macrophages and Dendritic cells) was statistically significant (*P* < 0.05, Fig. 8A**)**. Cumulative survive showed that B cells of immune infiltrates statistically significant (*P* < 0.05) of INHBA in HNSCC indicating that B cells significantly affecting the prognosis, it is worth further research and exploration Fig. 8B. Somatic copy number alterations (SCANs) are characterized by GISTIC 2.0, including deep deletion (−2), arm-level deletion (−1), diploid/normal (0), arm-level gain (1), and high amplification (2). Box plots are introduced to demonstrate the distributions of every immune subset at each copy number status with *INHBA* in HNSCC Fig. 8C. Finally, we contrast the INHBA expression between various tumor and normal tissue. The results showed that INHBA overexpression in bladder urothelial carcinoma (BLCA), colon adenocarcinoma (COAD), Head and Neck cancer (HNSC), and Liver hepatocellular carcinoma (LIHC) *et al*. (*P* < 0.05, Fig. 8D).

## Discussion

Differential expression and dysfunction of the TGF-β protein has been reported in various cancers^18,19^. INHBA encodes an individual from the TGF-β superfamily of proteins and the ligand could be further homo-dimerized to shape activin A or hetero-dimerized to frame inhibin with inhibin beta B^20^. In our previous bioinformatic analysis, we found that INHBA was overexpressed in HNSCC tissue based on online analysis tools. Until now, there is no effective biomarkers and therapeutic targets relevant to HNSCC progression and treatment response. So, it is emergency to identifying oncogenic biomarkers and elucidating the underlying mechanism of the initiation and development of HNSCC would make profound impact of the early diagnosis and effective treatment for patients with high malignancy^21^. To acquire more detailed information into the potential elements of INHBA in HNSCC and its regulatory mechanism, we performed bioinformatics analysis of open sequencing information to provide instruct future research in HNSCC.

Investigation of transcriptional sequencing information from thousands clinical samples TCGA databases comprising six geographic regions and subgroups analysis stratified based on gender, age, HPV status, gender, race and tumor grade confirmed that INHBA mRNA levels and CNVs are fundamentally higher in HNSCC when compare with normal tissue. Meanwhile, the fold change differences all were over 2 indicated strong that INHBA overexpression in HNSCC. We hypothesis that INHBA may be a potential diagnostic and prognostic marker and deserves further clinical validation. CNV affects gene expression through changes in gene sequence location and further influences disease susceptibility and disease progression^22^. High proportion of aberrant methylation being associated with cancer-associated CNAs while not somatically acquired epigenetic defects^23^. In our study, we found that mRNA upregulation is the most common alterations type of INHBA in HNSCC patients. This alteration may be caused by the overexpression of INHBA. We speculate that abnormal expression and dysfunction of INHBA result from protein modification and further cause changes in translation control and post-translation control. In addition, Kaplan-Meier survival analysis exhibited that high INHBA expression was related to overall survival and progression-free survival in HNSCC patients. The function of the identified neighbor genes of INHBA was involved in the activing binding, protein complex form, regulation of protein and several cancer processes. Thus, INHBA modifications is engaged with the center hub of post-transcriptional regulation, which is firmly related to protein translation.

Activins and inhibins assumed play a role in different physiological procedures through endocrine as well as autocrine or paracrine mechanisms^24^. They are also engaged with cell growth, proliferation, differentiation, apoptosis and carcinogenesis^25^. In order to start the activin cascade pathway, activin ties to a complex and triggers phosphorylation of the receptor and starts actuation of Smad proteins^26^. Studies revealed that high expression of INHBA gene was related to significantly poorer 5-year survival rate^27,28^. Previous studies have confirmed INHBA expression is related tumor invasiveness and promoting metastasis but the specific pathogenesis is unclear. Seder *et al*.^29^ found that INHBA was overexpressed and advanced cell proliferation via promoter demethylation and histone acetylation in esophageal adenocarcinoma. Kaneda *et al*.^30^ showed that activin A hinders vascular endothelial cell growth and further suppresses tumor angiogenesis in gastric cancer. Chang *et al*.^31^ also discovered that activin A advances tumor invasion and metastasis in HNSCC. Our study can provide information for further potential researches on regulatory mechanism. Using GSEA enrichment analysis of INHBA can help find vital networks of target kinases, miRNAs and transcription factors. Kinases and their related signaling pathways help balance and repair genomic DNA^32^. We found that INHBA in HNSCC is linked to a network of kinases including GRK3 and CSNK1E. GRK3 specifically phosphorylates activated beta-adrenergic and related G-protein coupled receptors. As an important signal regulator widely distributed in the body, GRK3 can keep cells de-sensitized during excessive signal stimulation^33^. It is reported in the literature that GRK3 is highly expressed in prostate cancer cells and can promote angiogenesis and distant metastasis of prostate cancer^34^. So we assume that INHBA and GRK3 play a synergistic role in the HNSCC. MiR-26b, is lowly expressed in various cancer tissues such as breast cancer and esophageal cancer, and participates in the growth process of tumor cells^35,36^. Jin *et al*.^37^ first demonstrated that miR-26a/b can advance apoptosis by means of suppressing the expression of autophagy initiator ULK1. He *et al*.^38^ indicated that miR-26 induced apoptosis and restrained autophagy based on the TGF-β1-JNK signaling pathway, recommending that miR-26 could be a useful novel target for the treatment of non-small cell lung cancer (NSCLC). We suggest that statement of miR-26a/b could induce apoptosis in HNSCC cells and needs more researches to convince. CP2 is a transcription factor that has a place with the Drosophila grainyhead-like gene family, and has been found to invigorate transcription of the a-globin gene^39^.In addition, CP2 have been implicated as being tumor suppressors in various human cancers. Despite their physiological importance, little is known about their structure and DNA binding mode^40^. In our study, we suggested that CP2 as a transcriptional factor that regulates by INHBA gene in light of HNSCC and further studies should test this theory.

Our study distinguished miRNAs that were related to INHBA using Targetscan online tool. To examine the function of the identified miRNAs, biological enrichment was performed via Funrich database. Biological processes were significantly enriched in regulation of nucleic acid metabolism; signal transduction; cell communication. Cell component were primarily enriched in the nucleus, cytoplasm, golgi apparatus. Molecular function was mainly enhanced in Transcription factor activity, transcription regulator activity. MiR-133 can significantly inhibited the migration and invasion of the cancer cells^41^. Study indicate that miR-153 affects the progression of nasopharyngeal cancer (NPC) by targeting the TGF-β2/Smad2 signaling pathway^42^. Overexpression of miR-140-3p inhibited cell proliferation, migration and invasion^43,44^. While dysregulation of these miRNAs of INHBA overexpression in HNSCC is extremely rare and need more researches to confirm. A superior comprehension of the interplay between the tumor microenvironment and the infiltrating immune cells is crucial. Characterization of the adaptive immune response has been appeared to be an indispensable prognostic tool in a wide scope of carcinomas, possibly significantly more important than the present cancer staging system^45,46^. In our Immune infiltrates study, cumulative survive showed that B cells of immune infiltrates statistically significant of INHBA in HNSCC indicating that B cells significantly affecting the prognosis and might have a prognostic significance, but it is worth further research and exploration.

This study gives staggered evidence for the significance of INHBA in head and neck squamous cell carcinoma and its potential role as a novel biomarker. Our outcomes recommend that INHBA overexpression in HNSCC has profound impacts in the center hub of post-transcriptional regulation, which is firmly related to protein translation. Meanwhile, we also examine the function of the identified miRNAs that were related to INHBA and molecular function of these miRNAs were mainly enhanced in transcription factor activity, transcription regulator activity. In addition, B cells of immune infiltrates affecting the prognosis and might have a prognostic significance related to INHBA in HNSCC. This study utilizes online tools dependent on the most prevalent bioinformatics theories to perform target gene analyses on tumor data information from open databases and empowers expansive scale HNSCC genomics research and subsequent functional explore^47^. Meanwhile, our study has limitations, one is that our results not verified in clinical samples and cannot provides precise clinical data, another is that due to the histological types and multiple anatomical sites and of HNSCC the tumor markers may vary widely. We will do a lot of related research in the follow-up.

## Methods

### We studied INHBA expression, mutations, regulation, function networks and immune infiltrates in data from patients with HNSCC based on different open databases by utilizing multi-dimensional analysis strategies

#### Oncomine analysis

The DNA copy number and mRNA expression of *INHBA* in HNSCC were investigated inside the Oncomine 4.5 database. Oncomine (www.oncomine.org) contains 715 gene expression data sets and 86,733 samples, is also the biggest oncogene chip database and incorporated data mining platform worldwide^48^. This analysis drew on a series of HNSCC studies, including Peng HNSCC, Sengupta HNSCC, Ginos HNSCC, Ye HNSCC, Pyeon HNSCC, and TCGA HNSCC studies^49–53^. *INHBA* expression was involved in evaluated in HNSCC tissue in respect to its expression in normal tissue, and *P* < 0.05 as the cutoff criterion considered statistically significant.

#### UALCAN analysis

UALCAN (http://ualcan.path.uab.edu) is a user-friendly, intelligence web asset for analyzing, integrating and discovering cancer transcriptome data and in-depth analyses of TCGA gene expression information^54^. TCGA uses large-scale sequencing-based genomic analysis technology to understand the molecular mechanisms of cancer through extensive collaboration. One of the portal’s highlights features is that it enables users to distinguish biomarkers or to perform in silico approval of potential genes of interest and evaluate gene expression in different tumor subgroups, such as gender, age, race, tumor grade *et al*.

#### c-BioPortal analysis

The cBioPortal (http://cbioportal.org)^55^ is an open-access asset gives visualization, analysis and download of substantial scale cancer genomics data sets which portal currently containing 245 cancer studies. We utilized c-BioPortal to analyze INHBA alterations in the TCGA HNSCC samples and shows an overview of genetic alterations per test in INHBA. Besides, using the Kaplan-Meier analysis in cBioPortal can evaluate the effect of gene expression dysregulation on the patient’s overall survival and disease-free survival. A tab biological interaction network of the INHBA and their co-expression genes was analyzed and neighboring genes with alteration frequencies were included. The Enrichr (https://amp.pharm.mssm.edu/Enrichr/) (version 6.8)^56^ which is a useful online platform database that incorporates biological information and gives a thorough set of functional annotation data of genes as well as proteins for users to analyze the functions or signaling pathways. So, we performed GO and KEGG pathway enrichment analyses of the most frequently modified neighbor genes using Enrichr. *P*-Value < 0.05 as statistically significant.

#### LinkedOmics analysis

LinkedOmics (http://www.linkedomics.org/ login.php)^57^ is openly accessible entry that includes multi-omics information from each of the 32 TCGA cancer. The LinkCompare module utilizes visualization functions and meta-analysis to look at and integrate affiliation results created by the LinkFinder module, which allows multi-omics analysis in a cancer type or pan-cancer analysis and enables user to scan for hopeful target genes of transcriptional factors, protein kinases or microRNAs. LinkedOmics was utilized to study genes differentially expressed related to INHBA in the TCGA HNSCC cohort (n = 520) and results were analyzed statistically using T-test then GSEA was utilized to perform analyses of GO and KEGG pathways analysis.

#### TargetScan analysis

TargetScan (http://www.targetscan.org/vert_72/)^58^ is a flexible web interface for predicts biological targets of miRNAs. TargetScanHuman considers matches to human 3′ UTRs and their orthologs, as characterized by UCSC whole-genome alignments. As an alternative, predictions are ranked by their likelihood of conserved targeting^59^. FunRich (http://www.funrich.org/)^60^ is an independent programming tool intended to deal with variety of gene/protein data sets regardless of the organism and for functional enrichment and interaction network analysis of genes and proteins. Currently, TargetScan was utilized to study miRNAs differentially expressed in connection with INHBA and then we used Funrich tool for miRNA enrichment analysis, including Biological process, Cellular component, Molecular function and Biological pathways.

#### TIMER analysis

TIMER (https://cistrome.shinyapps.io/timer/)^61^ is a comprehensive asset for systematical investigation of immune infiltrates over various malignancy types. The abundances of six immune infiltrates (CD8+ T cells, B cells, CD4+ T cells, Macrophages, Neutrphils and Dendritic cells) are assessed by our statistical method, which is approved using pathological estimations. This web server enables users to input function-specific parameters, with resulting figures dynamically showed to conveniently access the tumor immunological, clinical, and genomic features. Using Gene module to explore correlation between INHBA expression and abundance of immune infiltrates in HNSCC; Survival module to draw Kaplan-Meier plots for immune infiltrates and INHBA to picture the survival differences. We also provides the comparison of tumor infiltration levels among tumors with different somatic copy number alterations for INHBA in HNSCC. In addition, we explored the differential expression between tumor and normal tissues for INHBA over all TCGA tumors. *P*-Value < 0.05 was as statistically significant.

## References

[1] Xiao-Nan F (2018). Comprehensive analysis of competitive endogenous RNAs network associated with head and neck squamous cell carcinoma. Scientific Reports.

[2] Torre LA (2012). Global cancer statistics. CA Cancer J. Clin..

[3] Magnes T, Egle A, Greil R, Melchardt T (2017). Update on squamous cell carcinoma of the head and neck: ASCO annual meeting. Memo.

[4] Chin D (2005). Novel markers for poor prognosis in head and neck cancer. Int J Cancer.

[5] Guo-Fang G (2016). Overexpression of lncRNA H19/miR-675 promotes tumorigenesis in head and neck squamous cell carcinom. International Journal of Medical Sciences.

[6] Wu, Y. *et al*. SUZ12 is a novel putative oncogene promoting tumorigenesis in head and neck squamous cell carcinoma[J]. *Journal of Cellular and Molecular Medicine*, 10.1111/jcmm.13638 (2018). 10.1111/jcmm.13638 PMC6010759 29667751

[7] Reed, A. L. *et al*. High frequency of p16 (CDKN2/MTS-1/INK4A) inactivation in head and neck squamous cell carcinoma[J]. *Cancer Research*, **56**(16):3630–3633, 10.1002/(SICI)1097-0142(19960815)78:4<912::AID-CNCR31>3.0.CO;2-W (1996). 8705996

[8] Trivedi S, Mattos J, Gooding W, Godfrey TE, Ferris RL (2013). Correlation of tumor marker expression with nodal disease burden in metastatic head and neck cancer. Otolaryngol Head Neck Surg.

[9] Chen, Z. L., Qin, L., Peng, X. B., Hu, Y. & Liu, B. INHBA gene silencing inhibits gastric cancer cell migration and invasion by impeding activation of the TGF-β signaling pathway. *J Cell Physiol*., 10.1002/jcp.28439 (Apr 8 2019). 10.1002/jcp.28439 30963572

[10] Wang Q (2012). Upregulated INHBA expression is associated with poor survival in gastric cancer. Med. Oncol..

[11] Oshima T (2014). Relation of INHBA gene expression to outcomes in gastric cancer after curative surgery. Anticancer Res..

[12] Seder CW (2009). Upregulated INHBA expression may promote cell proliferation and is associated with poor survival in lung adenocarcinoma. Neoplasia.

[13] Yi J (2013). Clinical significance of angiopoietin-like 4 expression in tissue and the serum of esophageal squamous cell carcinoma patients. Medical Oncology.

[14] Hofland J (2012). Activin A stimulates AKR1C3 expression and growth in human prostate cancer. Endocrinology.

[15] Dean M, Davis DA, Burdette JE (2017). Activin A stimulates migration of the fallopian tube epithelium, an origin of high‐grade serous ovarian cancer, through non‐canonical signaling. Cancer Letters.

[16] Oshima T (2014). Relation of INHBA gene expression to outcomes in gastric cancer after curative surgery. Anticancer Research.

[17] Wang Q (2012). Upregulated INHBA expression is associated with poor survival in gastric cancer. Medical Oncology.

[18] Bose P, Rahmani M, Grant S (2012). Coordinate PI3K pathway and Bcl-2 family disruption in AML.[J]. Oncotarget.

[19] Torrealba, N. *et al*. TGF-β/PI3K/AKT/mTOR/NF-kB pathway. Clinicopathological features in prostate cancer. *Aging Male*., 1–11, 10.1080/13685538.2019, (Apr 11 2019).

[20] Taguchi, L *et al*. c-Ski accelerates renal cancer progression by attenuating transforming growth factor β signaling. *Cancer Sci*., 10.1111/cas.14018, (Apr 10 2019). 10.1111/cas.14018 PMC6550129 30972853

[21] Kang H, Kiess A, Chung CH (2015). Emerging biomarkers in head and neck cancer in the era of genomics. Nat Rev Clin Oncol.

[22] Jakobsson M (2008). Genotype, haplotype and copy-number variation in worldwide human populations. Nature.

[23] Martin-Trujillo A (2017). Copy number rather than epigenetic alterations are the major dictator of imprinted methylation in tumors. Nature Communications.

[24] Onagbesan OM (2004). Developmental changes in inhibin alpha and inhibin/activin betaA and betaB mRNA levels in the gonads during post-hatch prepubertal development of male and female chickens. Mol Reprod Dev.

[25] Chen YG (2006). Activin signaling and its role in regulation of cell proliferation, apoptosis, and carcinogenesis. Exp Biol Med.

[26] Lotinun S (2012). Activin receptor signaling: A potential therapeutic target for osteoporosis. Curr Mol Pharmacol.

[27] Okano M (2013). Significance of INHBA expression in human colorectal cancer. Oncol Rep..

[28] Katayama Y (2017). Clinical Significance of INHBA Gene Expression in Patients with Gastric Cancer who Receive Curative Resection Followed by Adjuvant S-1 Chemotherapy. Vivo.

[29] Seder CW (2009). Upregulated INHBA expression may promote cell proliferation and is associated with poor survival in lung adenocarcinoma. Neoplasia..

[30] Kaneda H (2011). Activin A inhibits vascular endothelial cell growth and suppresses tumour angiogenesis in gastric cancer. Br J Cancer..

[31] Chang WM (2016). Dysregulation of RUNX2/activin-A axis upon miR-376c downregulation promotes lymph node metastasis in head and neck squamous cell carcinoma. Cancer Res..

[32] Karimian A, Ahmadi Y, Yousefi B (2016). Multiple functions of p21 in cell cycle, apoptosis and transcriptional regula-tion after DNA damage. DNA Repair (Amst)..

[33] Taneja M (2011). Differential effects of inescapable stress on locus coeruleus GRK3 alpha 2-adrenoceptor and CRF1 receptor levels in learned helpless and non-helpless rats: a potential link to stress resilience. Behavioural Brain Research.

[34] Wenliang LI (2014). GRK3 is essential for metastatic cells and promotes prostate tumor progression. Proceedings of the National Academy of Sciences of the United States of America.

[35] Zhang ZH (2013). MicroRNA-26 Was Decreased in Rat Cardiac Hypertrophy Model and May Be a Promising Therapeutic Target. Journal of Cardiovascular Pharmacology.

[36] Li J (2014). MiRNA-26b inhibits cellular proliferation by targeting CDK8 in breast cancer. Int J Clin Experim Med.

[37] Jin F (2017). MiR-26 enhances chemosensitivity and promotes apoptosis of hepatocellular carcinoma cells through inhibiting autophagy. Cell Death and Disease.

[38] He, Y *et al*. miR-26 Induces Apoptosis and Inhibits Autophagy in Non-small Cell Lung Cancer Cells by Suppressing TGF-β1-JNK Signaling Pathway. *Front Pharmacol*., **9**:1509, 10.3389/fphar.2018.01509 (Jan 9 2019). 10.3389/fphar.2018.01509 PMC6333751 30687089

[39] Barnhart KM, Kim CG, Banerji SS, Sheffery M (1988). Identification and characterization of multiple erythroid cell proteins that interact with the promoter of the murine a-globin gene. Mol Cell Biol.

[40] Ming, Q *et al*. Structural basis of gene regulation by the Grainyhead/CP2 transcription factor family. *Nucleic Acids Research*, 10.1093/nar/gkx1299 (2018). 10.1093/nar/gkx1299 PMC5829564 29309642

[41] Xiao, B. *et al*. Expression of MicroRNA-133 Inhibits Epithelial–Mesenchymal Transition in Lung Cancer Cells by Directly Targeting FOXQ1. *Archivos de Bronconeumología (English Edition)*, S1579212916000185, 10.1016/j.arbr.2016.01.015 (2016). 10.1016/j.arbres.2015.10.016 26858166

[42] Guo, G *et al*. MicroRNA-153 affects nasopharyngeal cancer cell viability by targeting TGF-β2. *Oncol Lett*., **17**(1), 646–651, 10.3892/ol.2018.9570, (Jan 2019). 10.3892/ol.2018.9570 PMC6313159 30655812

[43] Liu, Y *et al*. MicroRNA-140 inhibits proliferation and promotes apoptosis and cell cycle arrest of prostate cancer via degrading SOX4. *J BUON*., **24**(1), 249–255, (Jan-Feb 2019). 30941977

[44] Zhou, Y. *et al*. miR-140-3p inhibits breast cancer proliferation and migration by directly regulating the expression of tripartite motif 28. *Oncol Lett*., **17**(4), 3835–3841, 10.3892/ol.2019.10038, (Apr 2019). 10.3892/ol.2019.10038 PMC6403497 30881504

[45] Galon J (2006). Type, density, and location of immune cells within human colorectal tumors predict clinical outcome. Science.

[46] Kawai O (2008). Predominant infiltration of macrophages and CD8(C) T Cells in cancer nests is a significant predictor of survival in stage IV nonsmall cell lung cancer. Cancer.

[47] Lin, Y *et al*. Expression and gene regulation network of RBM8A in hepatocellular carcinoma based on data mining. *Aging (Albany NY)*., **11**(2), 423–447, 10.18632/aging.101749, (Jan 22 2019). 10.18632/aging.101749 PMC6366983 30670676

[48] Rhodes DR (2007). Oncomine 3.0: genes, pathways, and networks in a collection of 18,000 cancer gene expression profiles. Neoplasia..

[49] Peng, C. H. *et al*. A Novel Molecular Signature Identified by Systems Genetics Approach Predicts Prognosis in Oral Squamous Cell Carcinoma. *Plos One*, 6, 10.1371/journal.pone.0023452 (2011). 10.1371/journal.pone.0023452 PMC3154947 21853135

[50] Sengupta S (2006). Genome-Wide Expression Profiling Reveals EBV-Associated Inhibition of MHC Class I Expression in Nasopharyngeal Carcinoma. Cancer Research.

[51] Ginos MA (2004). Identification of a gene expression signature associated with recurrent disease in squamous cell carcinoma of the head and neck. Cancer Research.

[52] Ye H (2008). Transcriptomic dissection of tongue squamous cell carcinoma. BMC Genomics.

[53] Pyeon, D. *et al*. Fundamental Differences in Cell Cycle Deregulation in Human Papillomavirus-Positive and Human Papillomavirus-Negative Head/Neck and Cervical Cancers *Cancer Res*., **67**(10), 4605–19. 10.1158/0008-5472.CAN-06-3619, 2007 May 15. 10.1158/0008-5472.CAN-06-3619 PMC2858285 17510386

[54] Chandrashekar, D. S. *et al*. Chakravarthi BVSK and Varambally S. UALCAN: A portal for facilitating tumor subgroup gene expression and survival analyses. *Neoplasia*., **19**(8), 649–658, 10.1016/j.neo.2017.05.002, (Aug 2017). 10.1016/j.neo.2017.05.002 PMC5516091 28732212

[55] Gao, *et al*. Sci. Signal. 2013 & Cerami *et al*. Cancer Discov. 2012 when publishing results based on cBioPortal, 10.1158/2159-829.

[56] Chen, E. Y. *et al*. Enrichr: interactive and collaborative HTML5 gene list enrichment analysis tool. *BMC Bioinformatics*., **128**(14), 10.1186/1471-2105-14-128 (2013). 10.1186/1471-2105-14-128 PMC3637064 23586463

[57] Vasaikar, S., Straub, P., Wang, J. & Zhang, B. LinkedOmics: analyzing multi-omics data within and across 32 cancer types. *Nucleic Acids Research*, gkx1090, 10.1093/nar/gkx1090 (2017). 10.1093/nar/gkx1090 PMC5753188 29136207

[58] Agarwal V, Bell GW, Nam J, Bartel DP (2015). Predicting effective microRNA target sites in mammalian mRNAs. eLife.

[59] Friedman RC (2008). Most mammalian mRNAs are conserved targets of microRNAs. Genome Research.

[60] FunRich: An open access standalone functional enrichment and interaction network analysis tool. *Proteomics*, **15**(15), 2597–2601, 10.1002/pmic.201400515 (2015). 10.1002/pmic.201400515 25921073

[61] Li T (2017). TIMER: A Web Server for Comprehensive Analysis of Tumor-Infiltrating Immune Cells. Cancer Research.

